# Percutaneous creation of direct intrahepatic portosystemic shunts – an alternative for failed TIPS creation

**DOI:** 10.1186/s42155-022-00292-4

**Published:** 2022-03-12

**Authors:** Karim Mostafa, Jens Trentmann, Julian Andersson, Rainer Günther, Felix Braun, Philipp Jost Schäfer

**Affiliations:** Universitätsklinikum Schleswig-Holstein, Campus Kiel, Kiel, Schleswig-Holstein Germany

**Keywords:** Direct intrahepatic portosystemic shunt, Percutaneous DIPS, Portal vein decompression

## Abstract

**Introduction:**

Direct intrahepatic portosystemic shunt creation is a feasible and safe alternative for transjugular intrahepatic portosystemic shunt creation. It needs equipment like endovascular ultrasound with restricted availability. We performed the procedure percutaneously with a common interventional armamentarium to make it more feasible.

**Methods:**

Retrospective analysis of 8 percutaneous DIPS insertions between 2016 and 2020.

**Results:**

The procedure was successful in 8/8 patients. There was no short-term death reported within 30 days. The longest reported patency is 5 years.

**Conclusion:**

Percutaneous DIPS creation is a feasible alternative for failed TIPS. Percutaneously the procedure can be completed faster than conventional DIPS using only minimal puncture equipment.

**Level of evidence:**

Level 4, Case Series.

## Introduction

Decompression of the portal vein via creation of a shunt to the hepatic venous system is an important therapeutic minimally invasive intervention for patients with decompensated liver cirrhosis suffering from portal hypertension with refractory ascites and/or uncontrollable upper GI-bleeding. (Angermayr et al., 2003) Under normal circumstances and suitable anatomic conditions the decompression is achieved by creation of TIPS. Other discussed indications for portal vein decompression are hydrothorax, portal hypertensive gastropathy, hepatorenal and hepatopulmonary syndromes as well as non-tumorous portal vein thrombosis. (Rajesh et al., 2020) However, due to the technical level of difficulty and potential pathoanatomic findings in liver cirrhosis, creation of TIPS is not always successful or even possible, and alternatives are limited. In 2001 an endovascular alternative, the DIPS, was published by Petersen et al., for which the use of endovascular ultrasound is mandatory. (Petersen et al., 2001) It has been reported as a feasible and safe endovascular alternative for TIPS insertion and is seen as a useful non-surgical method for portal vein decompression. (Ward et al., 2015; Peynircioglu et al., 2010; Petersen & Clark, 2008; Hoppe et al., 2008) Furthermore, the procedure is rarely used, as the largest reported case series by Hoppe et al. in 2008 included 18 patients. (Hoppe et al., 2008). Most DIPS are reported being created in an elective setting, with one study reporting DIPS creation in the setting of acute upper GI bleeding. (Ward et al., 2015) Our case series consists of 8 patients after failed TIPS creation, who underwent percutaneous creation of a DIPS between 2016 and 2020. With this research we aim to show that percutaneous creation of DIPS is a feasible alternative in patients where conventional TIPS creation is not possible.

## Material and methods

This study is an institutional review–board–approved, single-centre, retrospective analysis of all DIPS procedures performed between 2016 and 2020 at our institution. Eight patients, two women and six men, were included. Mean (range) age of the patients was 53.5 (16–79) years. All patients presented with liver cirrhosis with portal hypertension as underlying condition. Indication for percutaneous DIPS insertion was previous unsuccessful TIPS creation in three patients and pathoanatomic changes of the liver venous vasculature not allowing TIPS creation in five patients. All DIPS insertions were performed in an elective setting under general anaesthesia.

### Technique of percutaneous DIPS creation

**Fig. 1:**
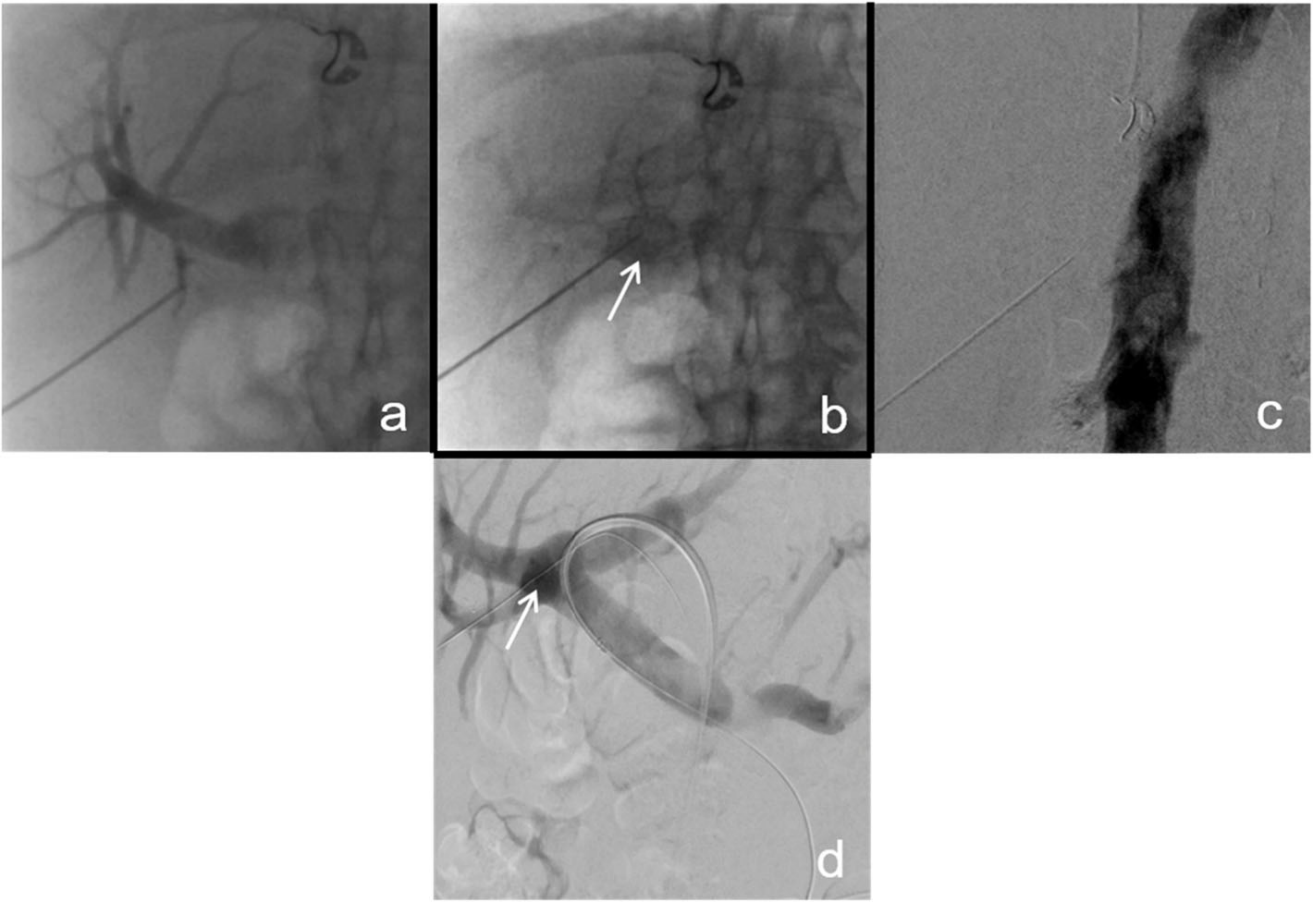
Fluoroscopic images of the percutaneous puncture of the main trunk of the right portal vein and the IVC. **A** In this case, initially a small branch of the portal vein was punctured as is seen in fluoroscopy. This was not a suitable vessel for DIPS creation. **B** The tip of the needle is in the main trunk of the right portal vein (white arrow image **B** and **D**), this is confirmed by angiogram. **C** The needle is further advanced into the IVC and correct positioning is confirmed in angiography. **D** Note the in place “buddy-wire” entering the right trunk of the portal vein via the initial puncture site (white arrow, image **B**)

**Fig. 2:**
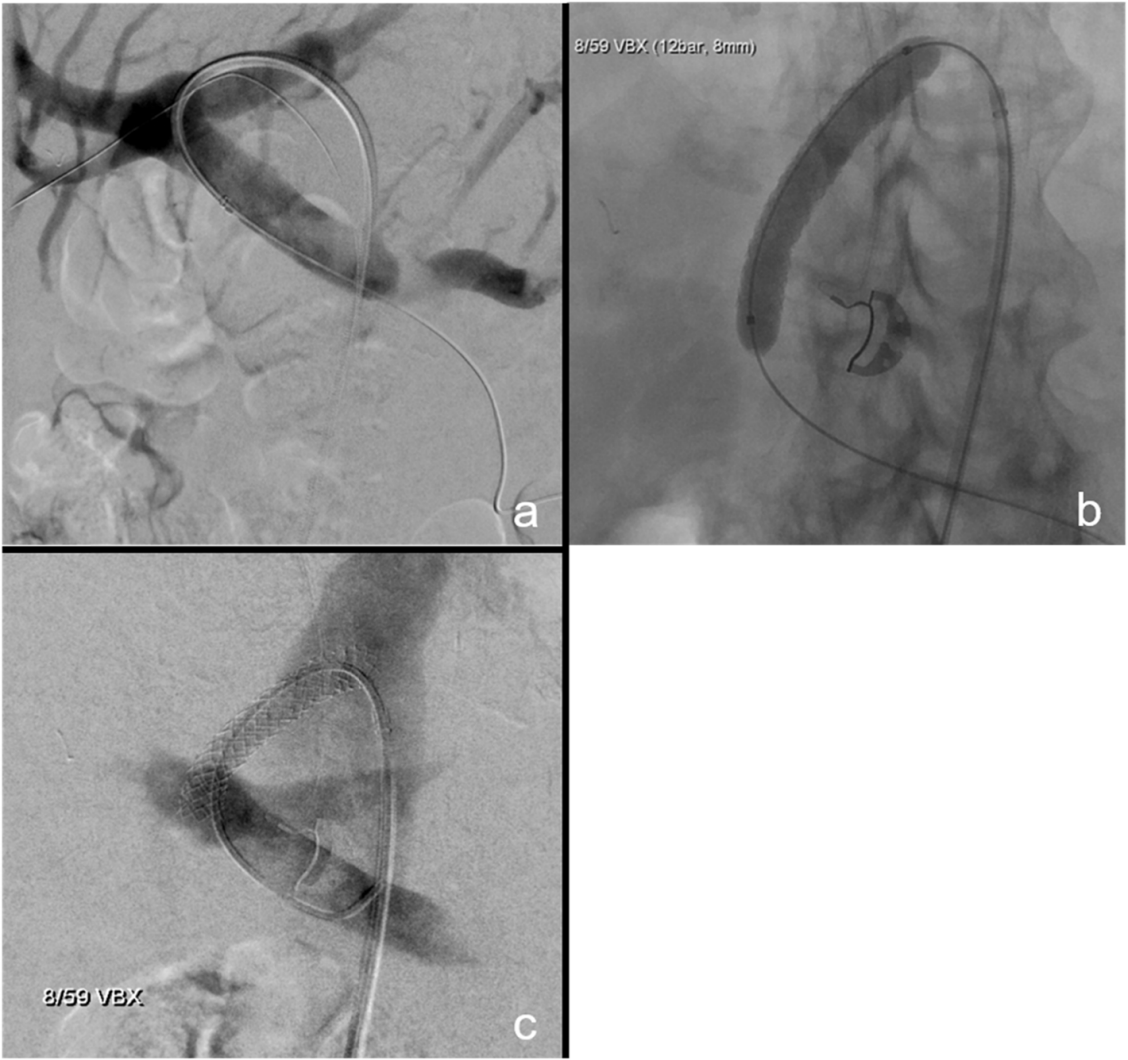
Final steps of DIPS creation. **A** After predilation, the 8-10F sheath is brought in via the inguinal venous access and is placed in the main trunk of portal vein. An angiogram is made to ensure correct positioning. **B** The “buddy wire” is removed and the balloon-inflatable 8 mm covered stent graft is brought in via the sheath. Care is taken to make sure that the covered parts of the endograft overlap its entrances into the portal vein and IVC. **C** A catheter is placed in the main trunk of the portal vein after creation of the DIPS tract and an angiogram is made to ensure correct positioning and patency

There are numerous ways reported on how to create a DIPS, on most occasions the use of endovascular ultrasound is described. (Ward et al., 2015; Petersen & Clark, 2008; Hoppe et al., 2008; Petersen et al., 2001). There are multiple factors to consider when performing this procedure (Table 1). Our procedural steps were as followed: Access to the main trunk of the right or left portal vein is percutaneously gained under ultrasound guidance with a 20G Chiba needle, interim confirming the needle’s position in the portal vein by angiogram (Fig. 1a, b). Next, the needle is further advanced through the portal vein into the inferior vena cava (IVC) through the caudate lobe of the liver, again confirming needle’s position by angiogram and sonography (Fig. 1c). At correct positioning a .018 in. guidewire (Radifocus Glidewire Advantage, Terumo, Tokyo, Japan) is placed in the IVC, then snared and externalised via a jugular or inguinal venous access. A 6F 45cm sheath (Destination, Terumo, Tokyo, Japan) is inserted in via the jugular/inguinal access and positioned in the IVC. Next, the parenchymal tract of the DIPS is predilated with a 2 mm balloon (Sterling, Boston Scientific, Marlborough/MA, USA) and the sheath is advanced over the inflated balloon into the portal vein. With the .018 in. buddy wire in place, a 4F catheter (Berenstein, Cordis, Miami Lakes/FL, USA) is brought in parallel to probe the portal vein and an .035 in. stiff wire (Amplatz, Boston Scientific) is placed (Fig. 2a). Afterwards the .018 in. buddy wire is removed. The 6F sheath is exchanged for an adequately sized sheath of 8-10F, depending on the endoprosthesis used for DIPS insertion, via the Amplatz wire in place. The new sheath is inserted into the main trunk of the portal vein. Afterwards the endoprosthesis is placed and deployed from the portal vein to the IVC for DIPS creation (Fig. 2b). A final angiography is performed to confirm position and patency (Fig. 2c). In our case series three patients received a dedicated TIPS endoprosthesis (Viatorr, Gore, Flagstaff/AZ, USA, size 10/60/20 mm), four a self-expanding endoprosthesis (Viabahn, Gore, sizes 2 × 10/50 mm, 1 × 8/50 mm and 1 × 10/100 mm), and one a balloon-expandable endoprosthesis (VBX, Gore, size 6/8/59 mm). Stent graft decision was based on measurements on preinterventional CT images and during fluoroscopy.

**Table 1:** Tips and Tricks for percutaneous ultrasound-guided DIPS creation

Tips and Tricks for DIPS procedures	Explanations
Drainage of ascites shortly before the procedure.	Excess amount of ascites may cause the liver to float within the abdomen subsequently making the puncture of the portal vein difficult. The position of the liver is also more stable when all ascites is drained.
Performing the procedure in general anesthesia.	A longer breath-hold can be achieved with the patient in general anesthesia - this makes especially the initial puncture of the portal vein and vena cava easier to perform.
Careful assessment of site, trajectory and angle of the puncture into the portal vein and the vena cava.	As this is the most important step of the procedure, it is of outmost importance to ensure that the main trunk of the right or left portal vein and subsequently the vena cava are punctured correctly through the caudate lobe of the liver.
Controlling the puncture of the portal vein and vena cava in sonography and fluoroscopy.	It is very important to keep the needle trajectory stable and to ensure a successful access to both vessels – this can be achieved by constant sonographic or fluoroscopic imaging during the puncture. The correct intravascular positioning has to be controlled by angiography once the needle is inside the portal vein and/or the vena cava.
Making sure that the covered parts of the endografts reach sufficiently into both the vena portae and vena cava before deploying them.	The covered parts of the endograft must cover the whole DIPS tract, and this can be achieved by letting the covered parts reach into the vena cava and vena portae. If this is not the case, there is a risk of free-lying uncovered retro- or intraperitoneal stent struts with subsequent risk of major bleeding.
Puncturing the portal vein with a stiff 18-20G needle and then continuing the puncture to the vena cava with a Chiba-needle in coaxial technique.	In order to facilitate access it can be helpful in some patients to perform the initial puncture of the portal vein with a stiff needle. Once access is gained, a Chiba-needle can be introduced coaxially and the vena cava is then punctured under sonographic and fluoroscopic imaging.

## Results

Successful DIPS insertion was achieved in 8/8 patients. In one case the first attempt of DIPS insertion failed but was successful one week later. We experienced no periinterventional complications. Median number of punctures until a successful DIPS was achieved was 1 (min. 1, max. 3). Two patients underwent revision of the DIPS within 4 months after insertion. DIPS dysfunction was diagnosed clinically, sonographically and in CT-angiography. Reasons for revision were stenosis of the DIPS outflow tract in both cases.

No deaths were reported within 30 days. Only short-term follow-up is available for two patients. One patient was lost to follow-up. Four patients were frequently controlled by ultrasound at our institution, and follow-up at 1.5, 2, 3 and over 5 years, respectively, confirmed excellent DIPS function.

## Conclusion and discussion

Our main findings are: 1) The percutaneous approach makes the technique wider feasible compared to conventional DIPS, as the creation of it can be achieved by using only minimal puncture equipment. 2) The percutaneous procedure can be performed faster than conventional DIPS insertion. The mean intervention time was 87 (range 48–135) minutes, which is faster than reported times for conventional DIPS insertion (Ward et al., 2015). 3) Despite anticoagulation management, two of our patients (25%) needed a revision due to DIPS outflow tract stenosis. Post-procedural DIPS dysfunction is a known complication in conventional DIPS, with primary patency rates of 75% within one year being reported in the literature. (Petersen & Binkert, 2004) This matches our findings. Both patients were successfully treated with balloon-angioplasty.

The literature shows that patients often undergo multiple TIPS creation attempts before DIPS is even considered, let alone attempted. (Ward et al., 2015; Peynircioglu et al., 2010; Hoppe et al., 2008) The need for endovascular ultrasound is likely contributing to the restricted use of DIPS. (Ward et al., 2015; Peynircioglu et al., 2010; Petersen & Clark, 2008; Hoppe et al., 2008; Petersen et al., 2001) Making DIPS insertion more feasible by our described percutaneous technique may open the door for a wider use of it as a valid alternative for patients in need of portal venous decompression and it may prevent a multitude of unsuccessful TIPS creation attempts. Therefore, based on the experience of our case series, we conclude that percutaneous DIPS is a valid alternative for failed TIPS insertion.

## Abbreviations

IVC: Inferior vena cava
DIPS: Direct intrahepatic portosystemic shunt
TIPS: Transjugular intrahepatic portosystemic shunt
